# Optimization of BSA-seq experiment for QTL mapping

**DOI:** 10.1093/g3journal/jkab370

**Published:** 2021-11-15

**Authors:** Likun Huang, Weiqi Tang, Weiren Wu

**Affiliations:** 1 Fujian Key Laboratory of Crop Breeding by Design, Fujian Agriculture and Forestry University, Fuzhou, Fujian 350002, China; 2 Institute of Oceanography, Marine Biotechnology Center, Minjiang University, Fuzhou, Fujian 350108, China

**Keywords:** BSA-seq, QTL, power, precision, influencing factor, experimental design

## Abstract

Deep sequencing-based bulked segregant analysis (BSA-seq) has become a popular approach for quantitative trait loci (QTL) mapping in recent years. Effective statistical methods for BSA-seq have been developed, but how to design a suitable experiment for BSA-seq remains unclear. In this paper, we show in theory how the major experimental factors (including population size, pool proportion, pool balance, and generation) and the intrinsic factors of a QTL (including heritability and degree of dominance) affect the power of QTL detection and the precision of QTL mapping in BSA-seq. Increasing population size can improve the power and precision, depending on the QTL heritability. The best proportion of each pool in the population is around 0.25. So, 0.25 is generally applicable in BSA-seq. Small pool proportion can greatly reduce the power and precision. Imbalance of pool pair in size also causes decrease of the power and precision. Additive effect is more important than dominance effect for QTL mapping. Increasing the generation of filial population produced by selfing can significantly increase the power and precision, especially from F_2_ to F_3_. These findings enable researchers to optimize the experimental design for BSA-seq. A web-based program named BSA-seq Design Tool is available at http://124.71.74.135/BSA-seqDesignTool/ and https://github.com/huanglikun/BSA-seqDesignTool.

## Introduction

Bulked segregant analysis based on deep sequencing (BSA-seq) is an efficient and cost-effective approach for rapid mapping of quantitative trait loci (QTLs). Since it was first reported in yeast (Ehrenreich *et al.* 2010), this approach has been widely applied to many different species especially in plants, such as rice (Arikit *et al.* 2019; Lei *et al.* 2020), wheat (Xin *et al.* 2020), tomato (Ruangrak *et al.* 2019), groundnut (Pandey *et al.* 2017), chickpea (Deokar *et al.* 2019), sunflower (Imerovski *et al.* 2019), squash (Ramos *et al.* 2020), watermelon (Branham *et al.* 2018), cricket (Pascoal *et al.* 2014), and Hessian fly (Navarro-Escalante *et al.* 2020). To facilitate BSA-seq for QTL mapping, a number of different statistical methods have been proposed, such as G′ test (Magwene *et al.* 2011), MULTIPOOL (Edwards and Gifford 2012), EXPLoRA (Duitama *et al.* 2014), Hidden Markov Model (Claesen and Burzykowski 2015), and Nonhomogeneous Hidden Markov Model (Ghavidel *et al.* 2015).

Recently, we proposed a new statistical method named block regression mapping (BRM) for BSA-seq (Huang *et al.* 2020). The method uses the simple and intuitional allele frequency difference (AFD) between two pools as statistic to test putative QTLs. Most importantly, it proves that smoothing by block regression can effectively remove the noise of sequencing (*i.e.*, the random error of resampling) and the expected AFD at a genomic position estimated by block regression is close to the actual AFD at the position even under very low sequencing depth. This means that the variation of AFD is basically determined by the size of the two pools. Based on this fact, the method reasonably resolves the problem of multiple testing correction in the estimation of significance threshold in BSA-seq, and can obtain both the point estimate and the 95% confidence interval (CI) of a QTL’s position. In addition, with the expected AFD of a QTL obtained by BRM, the proportion of variance explained by the QTL, or termed the QTL’s heritability, can be estimated using a method named Pooled QTL Heritability Estimater (PQHE; Tang *et al.* 2018).

Apart from the statistical method, experimental design is also very important for BSA-seq. An appropriate experimental design can effectively increase the statistical power of QTL detection and the precision of QTL mapping. At present, however, how to make a suitable experimental design for BSA-seq is still a problem to be solved. The size of population used in the BSA-seq experiments so far varies from very small (<100; Deokar *et al.* 2019; Imerovski *et al.* 2019) to very large (>10,000; Yang *et al.* 2013), with most being small (around 200; Das *et al.* 2014; Pandey *et al.* 2017; Branham *et al.* 2018; Luo *et al.* 2018; Arikit *et al.* 2019; Lahari *et al.* 2019; Ramos *et al.* 2020), and some medium (around 500; Xin *et al.* 2020) or large (near or over 1000; Ruangrak *et al.* 2019; Lei *et al.* 2020). The pool size used is also very diverse, varying from very small (only five individuals; Branham *et al.* 2018) to very large (>400; Yang *et al.* 2013), corresponding to a pool proportion (the ratio of pool size to population size) of ∼10% or less in most experiments.

In this paper, based on the principle of the BRM method, we investigate the factors that influence the power (of QTL detection) and the precision (of QTL mapping) in BSA-seq through theoretical derivation and numerical calculation as well as analysis on experimental data from yeast. According to the effects of these influencing factors, it is possible to optimize the experimental design for BSA-seq. 

## Materials and methods

### Experimental design for BSA-seq

There are various kinds of populations derived from a bi-parent cross (*P*_1_ × *P*_2_) for BSA-seq, including temporal populations such as F_*k*_ (*k *=* *2, 3, 4…) populations, and permanent populations such as recombinant inbred line (RIL) population, doubled haploid (DH) population, and haploid (H) population. F_*k*_ generation can be produced from F_*k*__−1_ generation either by selfing or by intercrossing (or random mating). These two different mating ways in F_*k*__−1_ generation will result in different genetic structures of the F_*k*_ population when *k *≥* *3. In this paper, we mainly analyze the situation of using the F_*k*_ population produced by selfing, and the term of F_*k*_ only refers to this type unless otherwise mentioned. Among the permanent populations, H population is usually used in fungi (*e.g.*, yeast), of which the life cycle is dominated by gametophyte generation. A pair of distinct DNA pools, namely, high-trait-value (H) pool *vs.* low-trait-value (L) pool (design A) or selected (S) pool *vs.* random (R) pool (design B), is established from the population and deeply sequenced. By mapping the sequencing reads to a reference genome, a very large number of molecular markers (mainly SNPs) and their counts in each pool can be found. QTL mapping is performed by comparing the two pools based on the marker data (Huang *et al.* 2020). In this paper, we shall focus on the optimization of design A. The principle should be also applicable to design B.

### Expectation of the power of QTL detection

Suppose a trait (*y*) is controlled by a QTL with two different alleles, *Q* from P_1_ and *q* from P_2_. The trait variation in an F_*k*_ or a permanent population can be described as a mixture distribution as below:

(1) $$f\left(y\right)=bf_{QQ}\left(y\right)+\left(1-2b\right)f_{Qq}\left(y\right)+bf_{qq}(y)=b\phi \left[\frac{y-(\mu -a)}{\sigma_{e}}\right]+\left(1-2b\right)\phi \left[\frac{y-(\mu +d)}{\sigma_{e}}\right]+b\phi \left[\frac{y-(\mu +a)}{\sigma_{e}}\right]$$

where a and d are the additive effect and dominance effect of the QTL, respectively; μ is the population mean; $\sigma_{e}$ is the standard deviation of the background (including genetic background and environment) variation; ϕ(·) is the probability density function of standard normal distribution; and $b=\left[1-{\left(1/2\right)}^{k-1}\right]/2$, where k→∞ in a permanent population.

Let $x=(y-\mu )/\sigma_{e}$, $a_{0}=a/\sigma_{e}$, and ${\;d}_{0}=d/\sigma_{e}$. Equation (1) can be rewritten as:

(2) fx=bϕx+a0+1-2bϕx-d0+bϕx-a0.

According to Equation (2), the additive effect heritability ($h_{a}^{2}$) and the dominance effect heritability ($h_{d}^{2}$) of the QTL are (Tang *et al.* 2018):

(3) ha2=2ba021+2ba02+2b1-2bd02

(4) hd2=2b1-2bd021+2ba02+2b1-2bd02.

And the total heritability of the QTL is: $h^{2}=h_{a}^{2}+h_{d}^{2}$. If the QTL does not exist, namely, the null hypothesis ($H_{0}:\;a_{0}=d_{0}=0$) is true, the mixture distribution $f\left(x\right)$ will degenerate into a standard normal distribution $\phi \left(x\right).$

Suppose the proportions of the H pool and the L pool in the population are $p_{H}$ and $p_{L}$, and the corresponding cut points for the H pool and the L pool are $x_{H}$ and $x_{L}$, respectively. According to Equation (2), we find:

(5) $$p_{H}=1-b\Phi \left(x_{H}+a_{0}\right)-\left(1-2b\right)\Phi \left(x_{H}-d_{0}\right)-b\Phi \left(x_{H}-a_{0}\right)$$

(6) $$p_{L}=b\Phi \left(x_{L}+a_{0}\right)+\left(1-2b\right)\Phi \left(x_{L}-d_{0}\right)+b\Phi \left(x_{L}-a_{0}\right),$$

where Φ(·) is the cumulative distribution function of standard normal distribution. Equations (5) and (6) indicate that $p_{H}$ and $p_{L}$ are determined when $x_{H}$ and $x_{L}$ are given and vice versa. According to Equations (2), (5), and (6), the allele frequency (AF, referring to the allele from *P*_1_) of the QTL in the H pool and that in the L pool are expected to be:

(7) $$\mu_{f_{H}}=\frac{1-2b\Phi \left(x_{H}-a_{0}\right)-(1-2b)\Phi \left(x_{H}-d_{0}\right)}{2p_{H}}$$

(8) $$\mu_{f_{L}}=\frac{2b\Phi \left(x_{L}-a_{0}\right)+(1-2b)\Phi \left(x_{L}-d_{0}\right)}{2p_{L}}.$$

Let $p=\left(p_{H}+p_{L}\right)/2$ and $\gamma =p_{H}/p_{L}$, we have $p_{H}=\;2p\gamma /\left(1+\gamma \right)$, and $p_{L}=\;2p/\left(1+\gamma \right)$. Thus, the AFD of the QTL between the two pools is expected to be:

(9) $$\mu_{\Delta f}=\mu_{f_{H}}-\mu_{f_{L}}=\frac{\left(1+\gamma \right)[1-2b\Phi \left(x_{H}-a_{0}\right)-\left(1-2b\right)\Phi \left(x_{H}-d_{0}\right)]}{4p\gamma }-\frac{\left(1+\gamma \right)\left[2b\Phi \left(x_{L}-a_{0}\right)+\left(1-2b\right)\Phi \left(x_{L}-d_{0}\right)\right]}{4p}.$$

According to the Central Limit Theorem, the sampled AFD will approximately follow a normal distribution with mean $\mu_{\Delta f}$ and variance

(10) $$\sigma_{\Delta f}^{2}=\sigma_{f_{H}}^{2}+\sigma_{f_{L}}^{2}=\frac{\mu_{f_{H}}\left(1-\mu_{f_{H}}\right)}{2^{t}p_{H}n}+\frac{\mu_{f_{L}}\left(1-\mu_{f_{L}}\right)}{2^{t}p_{L}n}=\frac{1+\gamma }{2^{t+1}p\gamma n}\left[\mu_{f_{H}}\left(1-\mu_{f_{H}}\right)+\gamma \mu_{f_{L}}\left(1-\mu_{f_{L}}\right)\right],$$

where n is the size of the mapping population; t=0 for permanent populations and t=1 for F_*k*_ populations. However, if the QTL does not exist ($H_{0}:\;a_{0}=d_{0}=0$), then $\mu_{f_{H}}=\mu_{f_{L}}=0.5$ and $\mu_{\Delta f}=0$ according to Equations (5)–(9). In this case, AFD will approximately follow a normal distribution with mean 0 and variance $\sigma_{0}^{2}$, where, according to Equation (10),

(11) $$\begin{array}{c} \sigma_{0}^{2}=\sigma_{\Delta f}^{2}\left(\mu_{\Delta f}=0\right)=\frac{{\left(1+\gamma \right)}^{2}}{2^{t+3}p\gamma n\;}. \end{array}$$

**Figure 1:**
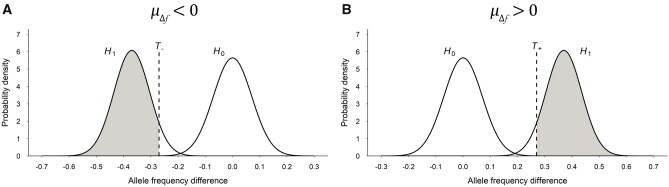
The power of QTL detection in BSA-seq using BRM. The AFD of the QTL follows a normal distribution with mean at AFD = 0 under the null hypothesis *H*_0_ (no QTL) or a normal distribution with mean at AFD = *µ*_Δ__*f*_ under the alternative hypothesis *H*_1_ (there is QTL). The power is equal to the shaded area (probability) under *H*_1_ on the left of the threshold *T*_−_ when *µ*_Δ__*f*_ < 0 (A) or on the right of the threshold *T*_+_ when *µ*_Δ__*f*_ > 0 (B).

So, the threshold of AFD at significance level α (two-tail test) is (Huang *et al.* 2020):

(12) T±=±uα/2σ0=±(1+γ)uα/22t+3pγn.

where the ± sign indicates the upper/lower threshold; $u_{\alpha /2}$ is the upper percentile point of α/2 in standard normal distribution, which is a constant for a species under a certain overall (genome-wise) significance level. Thus, the statistical power of detecting the QTL is expected to be (Figure  1):

(13) Power=1-ΦT+-μΔfσΔf+ΦT--μΔfσΔf.

### Expectation of the precision of QTL mapping

The mapping precision of a QTL can be indicated by the size of its CI. A narrower CI means a higher mapping precision. Consider a position M linked with a given QTL. No matter in an F_2_ or in an H/DH population, the AF at M in the H pool ($\mu_{f_{\mathrm{MH}}}$) and that in the L pool ($\mu_{f_{\mathrm{ML}}}$) are expected to be:

(14) $$\mu_{f_{\mathrm{MH}}}=\left(1-\theta \right)\mu_{f_{H}}+\theta \left(1-\mu_{f_{H}}\right)=\theta +(1-2\theta )\mu_{f_{H}}$$

(15) $$\mu_{f_{\mathrm{ML}}}=\left(1-\theta \right)\mu_{f_{L}}+\theta \left(1-\mu_{f_{L}}\right)=\theta +\left(1-2\theta \right)\mu_{f_{L}},$$

where *θ* is the recombination rate between M and the QTL. Therefore, the AFD at M between the two pools ($\mu_{\Delta f_{M}}$) is expected to be:

(16) $$\mu_{\Delta f_{M}}=\mu_{f_{\mathrm{MH}}}-\mu_{f_{\mathrm{ML}}}=\left(1-2\theta \right)\left(\mu_{f_{H}}-\mu_{f_{L}}\right)=\left(1-2\theta \right)\mu_{\Delta f}.$$

Equation (16) indicates that $\mu_{\Delta f_{M}}$ is a function of $\mu_{\Delta f}$ and θ, which describes the expected AFD curve around a QTL. It can be seen that $\mu_{\Delta f_{M}}$ varies between 0 (when θ=0.5, or M is far from the QTL) and $\mu_{\Delta f}$ (when θ=0, or M is just at the position of the QTL). Therefore, the AFD curve forms a positive peak (when $\mu_{\Delta f}>0$) or negative peak (when $\mu_{\Delta f}<0$) with the positive/negative top point being at the position of the QTL.

This AFD curve enables us to estimate the 95% CI (denoted as CI 95) of the QTL (Huang *et al.* 2020). Let $\mu_{\Delta f_{M}}=\mu_{\Delta f}-1.65\sigma_{\Delta f}$ (in the case of $\mu_{\Delta f}>0$) or $\mu_{\Delta f_{M}}=\mu_{\Delta f}+1.65\sigma_{\Delta f}$ (in the case of $\mu_{\Delta f}<0$). Substitute it into Equation (16), we find the recombination rate between the left (or right) border of CI 95 and the QTL:

(17) θ=0.825σΔfμΔf ,

where $\mu_{\Delta f}$ and $\sigma_{\Delta f}$ are determined by Equations (9) and (10), respectively. By assuming Kosambi’s mapping function and ignoring the influence of phenotypic selection on recombination rate (Lin and Ritland 1996), the corresponding genetic distance *D* (cM) would approximately be:

(18) D=25ln1+2θ1-2θ.

Therefore, the width of CI 95 of the QTL is 2*D* (cM).

For F_3_ and F_4_ populations, the parameter *θ* in Equations (14)–(17) should be replaced with $\theta_{3}$ and $\theta_{4}$, the apparent recombination rates in F_3_ and F_4_, respectively, where (Huang *et al.* 2020):

(19) $$\theta_{3}=\theta \left[1+\frac{1}{2}{\left(1-\theta \right)}^{2}\right]$$

(20) $$\theta_{4}=\theta \left[1+\frac{1}{2}{\left(1-\theta \right)}^{2}+\frac{1}{4}{\left(1-\theta \right)}^{4}\right].$$

From Equations (19) and (20), the real recombination rate *θ* can be calculated and thus the width of CI 95 can be calculated from Equation (18).

### Numerical analysis

Equations (13) and (18) describe the relationships of various factors (parameters) with the power of QTL detection and the precision of QTL mapping in BSA-seq using the BRM method. To display how the factors affect the power and precision, we used Equations (13) and (18) to analyze yeast H population (representing permanent populations) and rice F_2_, F_3_, and F_4_ populations (representing F_*k*_ populations), respectively. In the analyses, the value of $u_{\alpha /2}$ in Equation (12) under the genome-wise significance level of 0.05 for yeast H population and those for rice F_2_, F_3_, and F_4_ populations were 3.93 and 3.65, 3.74, and 3.78, respectively, of which the corresponding nominal significance level (α) was 8.49 × 10^−5^ and 2.62 × 10^−4^, 1.84 × 10^−4^, and 1.57 × 10^−4^, respectively (Huang *et al.* 2020). For simplicity, equal pool size (namely, γ=1 or $p_{H}=p_{L}=p$) was assumed except when the effect of pool size imbalance (*i.e.*, γ≠1 or $p_{H}\neq p_{L}$) was analyzed.

In addition, to demonstrate the influence of pool proportion in BSA-seq, we also used the BRM method (Huang *et al.* 2020) to analyze a series of simulated BSA-seq experiments based on the experimental data in an H population from yeast (Bloom *et al.* 2015). The data included the genotypes of 28,220 SNPs and phenotypes of trait GCS (end-point Growth on a medium containing Copper Sulfate) in 4276 segregants derived from a cross between a laboratory strain BY and a vineyard strain RM (Huang *et al.* 2020). We randomly extracted 4000 segregants from the total as the mapping population, from which a series of H *vs.* L pool pairs with different pool proportions (including 0.01, 0.05, 0.1, 0.2, 0.25, 0.3, 0.4, and 0.5) were made according to the GCS phenotype data. By dividing the genome into tandem 200-bp blocks, the actual AFD at every position (block) in the genome was calculated in each pool pair based on the SNP genotype data. The AFD thresholds at the genome-wise significance level of 0.05 were calculated according to the BRM method (Huang *et al.* 2020). It was considered that a QTL existed when the maximum AFD value in a peak region exceeded the threshold.

## Results

### Effect of population size

**Figure 2:**
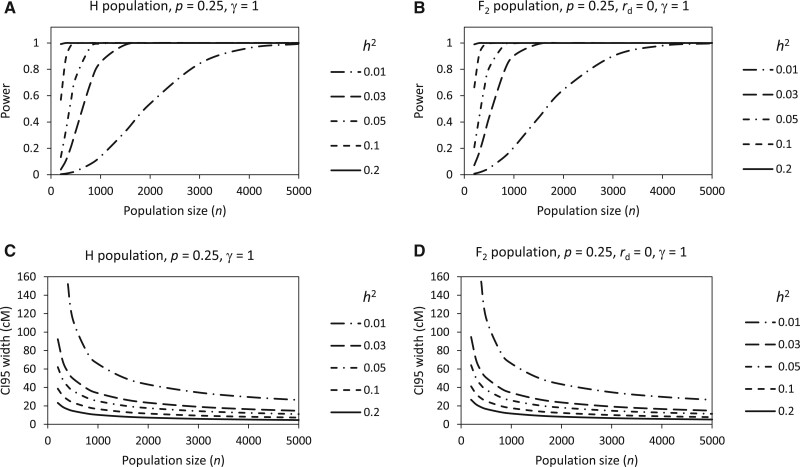
Relationships of power and CI 95 width with population size depending on QTL heritability in yeast H population (A, C) and rice F_2_ population (B, D).

Population size (*n*) and pool proportion (*p*) are two major experimental factors affecting the power and precision in BSA-seq. When *p* is fixed, depending on the QTL heritability (*h*^2^), the power and the CI 95 width display a series of S-shape curves (Figure  2, A and B) and L-shape curves (Figure  2, C and D) with *n*, respectively. So, approximately, the process of power increase along with the *n* increase could be divided into three stages: slow→fast→slow, while the process of CI 95 width decrease along with the *n* increase could be divided into two stages: fast→slow. Obviously, increasing *n* in the last stage is inefficient for power increase and CI 95 width decrease. This suggests that a value of *n* just before the start point of the last stage would be optimal. However, different QTL may have different optimal *n*, which is inversely proportional to *h*^2^ (Figure  2). A larger *h*^2^ would have a smaller optimal *n*.

### Effect of pool proportion

**Figure 3:**
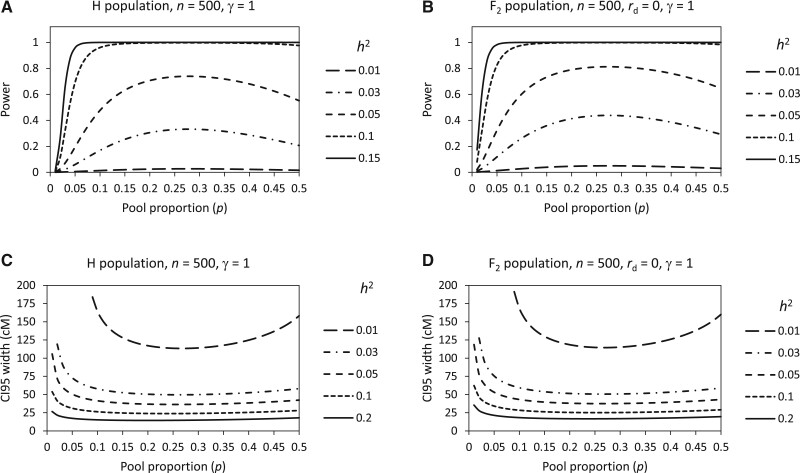
Relationships of power and CI 95 width with pool proportion depending on QTL heritability in yeast H population (A, C) and rice F_2_ population (B, D).

Theoretically, *p* varies between 0 and 0.5. The extreme situation *P = *0.5 means that the population is divided into two pools of equal size just at the mid-point of the trait. When *n* is fixed, it is seen that the power (Figure  3, A and B) and the CI 95 width (Figure  3, C and D) are neither a monotonic function of *p*. There is a peak of power and a valley of CI 95 width, respectively. The highest point of power is mainly located between *P = *0.25 and *P = *0.3, while the lowest point of CI 95 width is located around *P = *0.25, depending on *h*^2^. This suggests that 0.25 is a generally suitable, if not the best, value of *p*. Nonetheless, the peak top of the power and the valley bottom of the CI 95 width are broad and flat, especially when *h*^2^ is large. Therefore, the suitable value of *p* can be flexible in a wider range. It is noticeable that small *p* has very strong unfavorable effects on the power and the CI 95 width. As *p* decreases toward zero, the power will quickly drop toward zero and the CI 95 width will soar toward infinite, no matter how large *h*^2^ is. Therefore, it is inappropriate to use small *p*.

### Effect of interaction between population size and pool proportion

**Figure 4:**
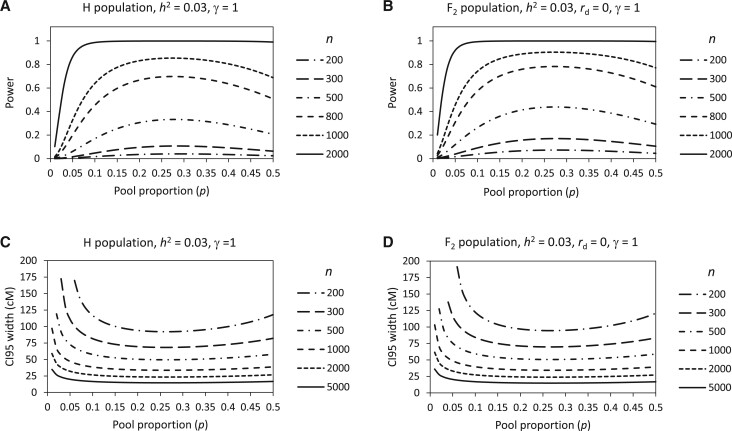
Relationships of power and CI 95 width with pool proportion depending on population size in yeast H population (A, C) and rice F_2_ population (B, D).

When the intrinsic factor *h*^2^ is fixed, it is seen that the basic feature of the relationship between the power and *p* and that between the CI 95 width and *p* (Figure  3) remain the same under different *n* (Figure  4). The value of *P = *0.25 still appears to be generally suitable (Figure  4). However, the effects of *p* on the power and the CI 95 width are related to *n*. As *n* increases, the peak top of the power and the valley bottom of the CI 95 width will become wider and flatter. Therefore, the suitable value of *p* can be more flexible under larger *n*. It is noted that the peak top of the power also becomes flatter when *n* is very small (Figure  4, A and B). But in this case, the power is very low and therefore is meaningless.

### Effect of pool imbalance

**Figure 5:**
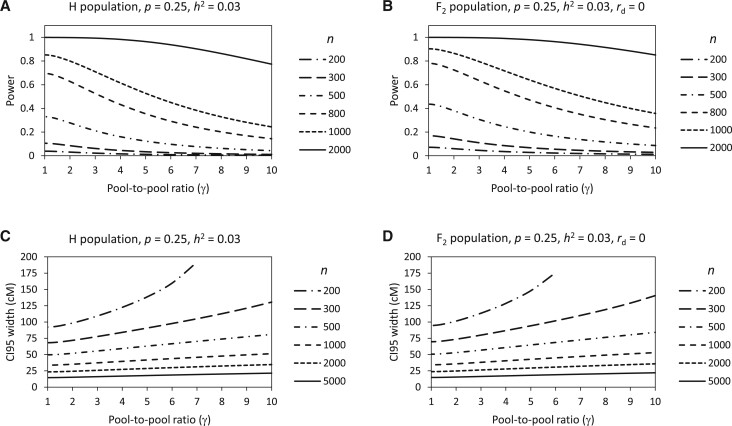
Relationships of power and CI 95 width with pool-to-pool ratio depending on population size in yeast H population (A, C) and rice F_2_ population (B, D). Only the case of γ ≥ 1 is shown because *p*_H_/*p*_L_ < 1 is equivalent to *p*_L_/*p*_H_ > 1.

In the above analyses, it is assumed that the two pools are balanced in size, namely, γ=1 or $p_{H}=p_{L}=p$. By fixing *p* and *h*^2^, it is found that imbalance of pool size (γ≠1) can reduce the power (Figure  5, A and B) and increase the CI 95 width (Figure  5, C and D). The more the γ deviates from 1, the stronger the effect of pool imbalance, but increasing *n* can attenuate the effect of pool imbalance to some extent. The result suggests that the optimal design is to use two pools of equal size.

### Effects of degree of dominance and generation

**Figure 6:**
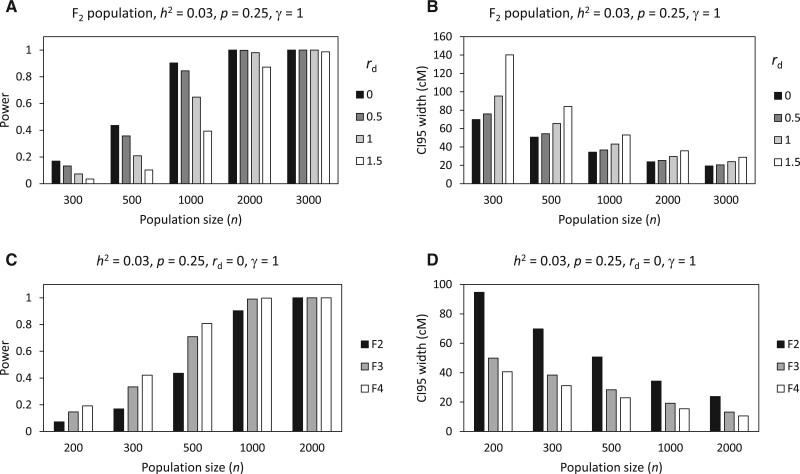
Relationships of power and CI 95 width with degree of dominance (A, B) and generation (C, D) depending on population size in rice.

In F_*k*_ populations, the dominance effect of a QTL may exist and therefore affect the result of BSA-seq. In the above analyses, it is assumed that there is no dominance effect (namely, the degree of dominance *r*_d_ = 0) in the F_2_ population. However, if *r*_d_ > 0 but *h*^2^ is fixed, the power will be reduced (Figure  6A) and the CI 95 width will be increased (Figure  6B). The larger the *r*_d_ is, the smaller the power and the larger the CI 95 width will be. This suggests that additive effect is more beneficial than dominance effect to QTL mapping in BSA-seq.

Unlike permanent populations, F_*k*_ populations have different genetic structures in different generations. This can affect the result of BSA-seq. When other conditions are the same, the power increases (Figure  6C) and the CI 95 width decreases (Figure  6D) as the generation increases. The increment of power and the decrement of CI 95 width are particularly significant from F_2_ to F_3_. However, the power increase is attenuated or even disappeared when the population is large, while the CI 95 decrease remains significant (the relative decrease from F_2_ to F_3_ is always ∼50%) with little influence by the population size.

### Simulation of BSA-seq based on experimental data from yeast

**Figure 7:**
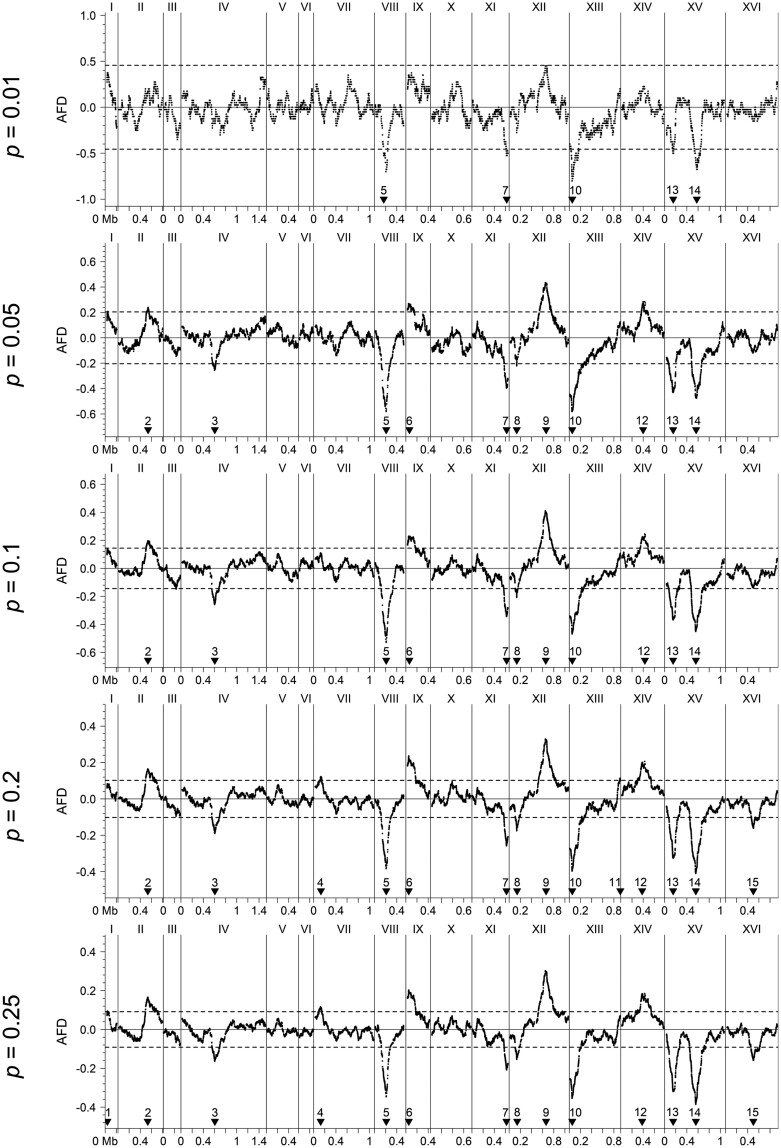

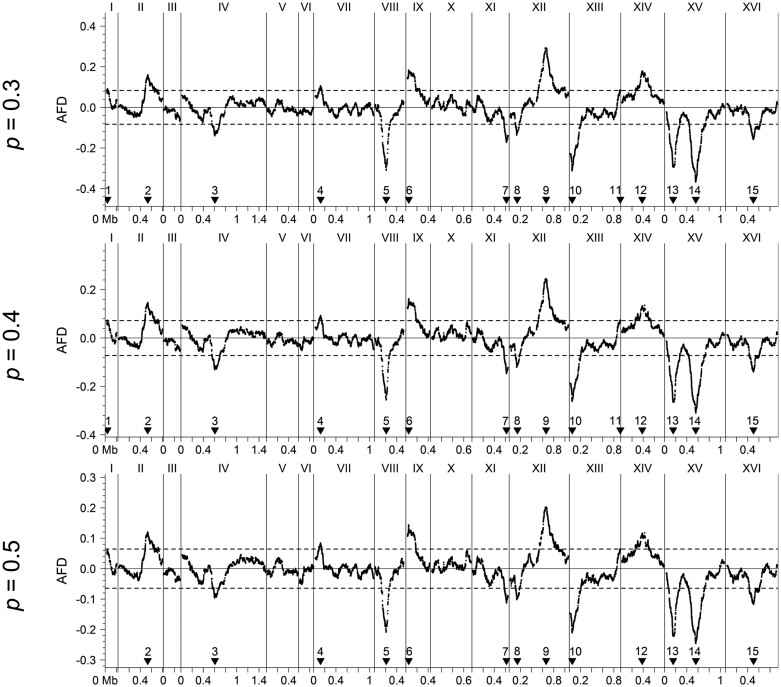
Actual AFD profiles under different pool proportions in a real yeast H population consisting of 4000 strains. The horizontal dashed lines are thresholds at the genome-wise significance level of 0.05. The black filled reversed triangles indicate the positions of detected QTLs, which are numbered from left to right.

To demonstrate the effect of pool proportion in practical populations, we used a set of experimental data from yeast (Bloom *et al.* 2015) to simulate BSA-seq using different pool proportions. The results were consistent with the theoretical expectation (Figure  7). A total of 15 putative QTLs were detected, with 5, 11, 11, 14, 14, 15, 15, and 13 QTLs detected under the pool proportion of 0.01, 0.05, 0.1, 0.2, 0.25, 0.3, 0.4, and 0.5, respectively. The number of detected QTLs was greatly reduced when *p* was small, but basically remained stable when *P *≥* *0.2, varying only at two QTLs with small peaks (QTL1 and 11), which were marginally significant (just reaching or slightly exceeding the threshold) at the maximum.

## Discussion

We have shown above the effects of several factors, including experimental factors (population size, pool proportion, pool balance, and generation) and intrinsic factors (QTL heritability and degree of dominance), on the power of QTL detection and the precision (CI 95 size) of QTL mapping in BSA-seq in two different types of populations, H population (yeast) and F_2_ population (rice). The factors display similar relationships with the power and CI 95 size in the two types of populations (Figures  2–5), suggesting that the laws of the relationships revealed in this study are universal in BSA-seq. The intrinsic factors are mainly determined by the characteristics of QTLs, while the experimental factors can be managed in experimental design. Hence, according to the effects of various experimental factors, it is able to optimize the experimental design for BSA-seq.

Generally speaking, the experimental design for BSA-seq mainly involves three aspects, namely, population type (what kind of population), population size, and pool proportion. Population type is not an issue for fungi because only H population is applicable. For plants, however, there are multiple choices, such as F_*k*_ population, RIL population, and DH population. Among them, the most convenient type is probably F_2_ population. However, we have seen that higher F_*k*_ generations are better for the power and precision (Figure  6, C and D). This is understandable because the frequency of homozygous genotypes of a QTL will increase and more recombination events between the QTL and flanking markers will occur as the generation increases. The former will likely increase the AFD between the two pools and thus increase the power because additive effect generally contributes more to genetic variation than dominance effect; the latter will increase the resolution of mapping and thus reduce the CI 95 size. As the improvements of power and precision are the most significant from F_2_ to F_3_ (Figure  6, C and D), it is recommended using F_3_ instead of F_2_ in practice if time permits. Nonetheless, it is necessary to point out that for the type of F_*k*_ population produced by random mating instead of selfing, the higher generations (*k *≥* *3) all have the same population structure as F_2_ in terms of a single locus. In this case, the power does not increase with generation. Instead, the power may decrease with generation because the significance threshold of AFD increases with generation (Huang *et al.* 2020).

According to the principle of BSA (Michelmore *et al.* 1991), the smaller the pool proportion is, the greater the difference between the two pools will be. However, studies of conventional BSA method by theoretical analysis based on the infinitesimal model (Gallais *et al.* 2007) and computer simulation (Navabi *et al.* 2009) and simulation study of BSA-seq based on G′ test (Magwene *et al.* 2011) all show that the power of QTL detection reach the maximum value not under small pool proportions but under larger ones. This was verified in our study (Figures  3, A and B4, A and B). The reason is that the power is determined not only by the AFD between the two pools but also by the variance of AFD. Decreasing pool proportion can increase AFD and its variance simultaneously, which will make the power increase and decrease, respectively. When the effect of AFD increase is smaller than the effect of AFD variance increase due to the decrease of pool proportion, the power will decrease. Apart from the effect on power, we also analyzed the effect of pool proportion on the CI 95 size (Figures  3, C and D4, C and D), revealing that pool proportion affects the power and the CI 95 size correspondingly. So, considering the power and the CI 95 size simultaneously, we found that a pool proportion of 0.25 is generally suitable for BSA-seq. Nonetheless, pool proportion can be flexible in a wide range when the population size is large.

Intuitively, the two pools are usually set to be equal or balanced in size in BSA-seq. However, the benefit of pool balance has not been studied. In some studies, the two pools are very different in size. For example, in a BSA-seq experiment for mapping QTLs underlying the high ethanol tolerance in yeast, the two pools consisted of 32 and 237 segregants, respectively (Pais *et al.* 2013). In this study, we proved that imbalance of pool size is harmful, especially when the difference between the two pools is large, which can reduce the power and increase the CI95 size significantly (Figure  5). Hence, pool balance is important.

We have shown that increasing population size can increase the power and reduce the CI95 size under a constant pool proportion, but the improvement of power and CI95 size due to population size increase is very small when the population is sufficiently large (Figure  2). In practice, population size is also restricted by the cost of phenotyping. So, it is not that the larger the population, the better. However, determining the suitable population size is not easy because it depends on the heritability of each QTL. We suggest that the suitable population size can be chosen in light of a typical minor QTL (*e.g.*, *h*^2^ = 0.03). It can be seen from Figure  2 that in both yeast H population and rice F_2_ population, 1500 can be considered to be a suitable population size for a QTL with *h*^2^ = 0.03, at which the power basically reaches 100% and the CI95 size has been within the slow decrease stage. In practice, researchers may want to know what the minimum population size is needed to detect a QTL with a given or higher heritability at a required power, or what the power is expected for a QTL with a given heritability when the population size is fixed. To meet these needs, we developed a web-based tool named BSA-seq Design Tool, which can be visited at http://124.71.74.135/BSA-seqDesignTool/ or downloaded from https://github.com/huanglikun/BSA-seqDesignTool. The tool will facilitate researchers to optimize their experimental designs of BSA-seq for QTL mapping.

## Data availability

A web-based program named BSA-seq Design Tool is available at http://124.71.74.135/BSA-seqDesignTool/ and https://github.com/huanglikun/BSA-seqDesignTool.

## Funding

This study was supported by the Sci-Tech Innovation Special Fund of Fujian Agriculture and Forestry University (grant number CXZX2017248). 

## Conflict of interest

The authors declare that there is no conflicts of interest.
